# Lessons learned from a textbook outbreak: EHEC-O157:H7 infections associated with the consumption of raw meat products, June 2012, Limburg, Belgium

**DOI:** 10.1186/2049-3258-72-44

**Published:** 2014-12-15

**Authors:** Toon Braeye, Sarah Denayer, Klara De Rauw, Anmarie Forier, Jurgen Verluyten, Ludo Fourie, Katelijne Dierick, Nadine Botteldoorn, Sophie Quoilin, Pascale Cosse, Jeannine Noyen, Denis Pierard

**Affiliations:** Scientific Institute of Public Health, Department Epidemiology, Epidemiology of infectious diseases, Juliette Wytsmansstraat 14, 1050 Brussels, Belgium; Scientific Institute of Public Health, Scientific service of foodborne Pathogens, NRL VTEC in Food-NRL Foodborne Outbreaks, Juliette Wytsmansstraat 14, 1050 Brussels, Belgium; Department of Microbiology, NRC STEC/VTEC, Universitair Ziekenhuis Brussel, Vrije Universiteit Brussel (VUB), Laarbeeklaan 101, 1090 Brussels, Belgium; Department of Infectious Disease Control, Agency for Care and Health, Koningin Astridlaan 50, 3500 Limburg, Belgium; Federal Agency for Safety of the Food Chain, FASFC, Kruidtuinlaan 55, 1000 Brussel, Belgium

**Keywords:** Enterohaemorrhagic E. coli – EHEC, Escherichia coli O157, Food-borne infections, Vero cytotoxin-producing E. coli – VTEC, Outbreaks, Case control studies, PFGE, IS629-printing

## Abstract

**Background:**

On 5 June 2012 several enterohemorrhagic *Escherichia coli*, EHEC, O157:H7 infections were reported to the public health authorities of Limburg.

**Methods:**

We performed a case-control study, a trace back/forward investigation and compared strains isolated from human cases and food samples. A case was defined as anyone with a laboratory-confirmed *E. coli* O157:H7-infection in North-East Limburg from May 30 2012 till July 15 2012. Family members with bloody diarrhea were also included as cases. *E. coli* O157 was isolated by culture and the presence of the virulence genes was verified using (q)PCR. Isolates were genotyped and compared by Pulsed Field Gel Electrophoresis (PFGE) and insertion sequence 629-printing (IS629-printing).

**Results:**

The outbreak involved 24 cases, of which 17 were laboratory-confirmed. Five cases developed Hemolytic Uremic Syndrome (HUS) and fifteen were hospitalized. Cases reported a significantly higher consumption of “steak tartare”, a raw meat product (OR 48.12; 95% CI; 5.62- 416.01). Cases were also more likely to buy meat-products at certain butcheries (OR 11.67; 95% CI; 1.41 - 96.49). PFGE and IS629-printing demonstrated that the *vtx1a vtx2a eae ehxA* positive EHEC O157:H7 strains isolated from three meat products and all seventeen human stool samples were identical. In a slaughterhouse, identified by the trace-back investigation, a carcass infected with a different EHEC strain was found and confiscated.

**Conclusion:**

We present a well described and effectively investigated foodborne outbreak associated with meat products. Our main recommendations are the facilitation and acceleration of the outbreak detection and the development of a communication plan to reaches all persons at risk.

**MESH:**

Foodborne diseases, Shiga-toxigenic Escherichia coli, Enterohemorrhagic Escherichia coli, Meat products, Case control studies, Electrophoresis, Gel, Pulsed-Field

**Electronic supplementary material:**

The online version of this article (doi:10.1186/2049-3258-72-44) contains supplementary material, which is available to authorized users.

## Background

Enterohemorrhagic *Escherichia coli,* EHEC, (also called Verotoxin-producing *E. coli*, VTEC, or Shiga-like toxin-producing *E. coli,* STEC, associated with hemorrhagic colitis) is known to easily spread via food and cause infections with symptoms ranging from mild watery diarrhea to hemorrhagic colitis and hemolytic uremic syndrome, HUS [1]. In 2011, 12 EU countries reported 63 foodborne outbreaks, caused by EHEC strains [2]. The most commonly associated serogroup is O157 [3, 4]. The first published large outbreak with *E. coli* O157:H7 occurred in 1982 in the United States and involved contaminated hamburgers. The transmission of *E. coli* O157 occurs primarily by ingestion of inadequate processed contaminated food or water, but also through contact with manure or animals at farms and petting zoos [5, 6]. Person to person transmission has been described in daycare centers, nurseries and certain institutions [7].

In Belgium, governmental departments, laboratories and the scientific institute of public health, IPH, are collaborating for the prevention and management of outbreaks. Three National Reference Laboratories, NRL, for VTEC are officially recognized; one for human samples (National Reference Center, NRC), one for food samples (National Reference Laboratory for Foodborne Outbreaks ,NRL-FBO) and one for veterinary samples. The IPH hosts the NRL-FBO, organizes the network of the human reference centers and offers epidemiological support during outbreaks [8]. The Federal Agency For the Safety of the Food Chain, FASFC, is concerned with the safety along the food chain; hygiene inspection, sampling and it manages the system for tracing food products back and forward [9]. Notification of foodborne outbreaks to the local public health authority is mandatory in the Flemish region of Belgium; two or more cases of foodborne disease, linked or possibly linked, should be reported by clinicians or laboratories.

*E. coli* O157 was first described in Belgium in 1987. From 1994 to 2011, between 47 and 103 EHEC cases were registered yearly in Belgium. The most frequently detected serotype was O157, 24.1% of the EHEC positive stools was *E. coli* O157:H7/H*-*[10, 11]. The NRC detected an association between *E. coli* 0157 and HUS. In 2011 twelve EHEC strains were detected in HUS cases, ten of these were serotyped as *E. coli* O157:H7. In 2012 all O157-isolates tested positive for the virulence factors intimin (*eae*) and enterohemolysin (*ehxA*). Intimin is associated with the formation of “attachment-effacement”-lesions and enterohemolysin is a plasmid-encoded toxin.

Previously two Belgian outbreaks with a microbiologically confirmed link between human cases and the origin of the EHEC infection have been published. One outbreak in 2006 with only 2 cases, of which none developed HUS, could be linked to contact with farm animals [12]. A larger outbreak in 2007 with 12 cases, of which five developed HUS, could be linked to ice cream contaminated with *E. coli* O26 and *E. coli* O145 [13].

On June 5^th^ 2012 two cases diagnosed with an EHEC-infection were reported to the department of public health of Limburg. One day earlier another case had already been reported. We describe the further course of this outbreak and the investigation towards the source.

## Methods

### Epidemiology

#### Case finding & exploratory interviews

The outbreak was detected and the cases were identified through the system of mandatory reporting. The public health authority contacted local general practitioners and hospitals to inform them on the outbreak and ask them to report additional cases. The public health authority also contacted all cases telephonically for an interview. Information on personal characteristics (age, gender), family size, symptoms in family members, possible sources, diet history, food preferences and in which shops they recently bought food products was collected.

#### Case-control study

A case in the case-control study was defined as anyone with a laboratory-confirmed *E. coli* O157:H7 infection in the North-East Limburg region from May 30 2012 until June 14 2012, the start of the case-control study. Out of the 13 laboratory-confirmed cases known by the start of the case-control study, we randomly selected eleven cases and invited them to an electronic survey. The two cases that were not selected, provided the information necessary to compose a list of suspicious supermarkets for the survey. Two hundred persons living in the direct neighbourhood of the cases were invited to the same electronic survey. A standard survey is available for case-control studies in foodborne outbreaks. As the exploratory interviews, conducted by the start of the case-control study, pointed towards meat products, we shortened this survey by excluding specific questions about diary, vegetable and fruit consumption. The final survey included 13 meat products, three dairy products (including ice-cream) and six other food products (vegetables and sprouts). The survey also included questions on outdoor dining at a restaurant or snack bar and shopping at a supermarket in a list of five supermarkets. Finally the survey gathered information on symptoms and the onset date of these symptoms. The persons who completed the survey and did not report any symptoms were considered controls.

In the univariate analysis we calculated unadjusted odds ratios. Logistic regression was used for multivariate analysis. Variables attaining a significance level of p < 0.2 in univariate analysis were included in multivariate analysis. Overall, a significance level of p < 0.05 was set as statistically significant. Analysis were performed using SAS software (version 9.3, SAS Institute Inc., Cary, NC, USA).

### Microbiology

In Belgium, beef meat was traceable back and forward through the use of unique “Sanitel-numbers” and data recorded in a computerized management system. Cattle could be followed through an individual identification number from farm through slaughterhouse, cutting plant, butcheries into retail. Once suspicion had risen on a meat product, samples were taken from the complete production and distribution chain associated with this product. All data concerning sampling is encoded in the computer system ‘Foodnet’ of FASFC. Samples were analyzed at the NRL-FBO IPH. Human samples were further analyzed at the NRC UZBrussel.

For the detection of EHEC in stool, an in-house multiplex PCR was used on cultures of clinical samples. Initial typing of EHEC isolates was done using biochemical tests, in-house PCR and agglutination. Subtyping of the *vtx* genes was done using a PCR method described by Scheutz et al. [14].

For the detection in and isolation of EHEC from meat and carcass samples, three detection and/or isolation methods were used in parallel, including the recently published ISO/TS 13136:2012 for the detection of EHEC, ISO 16654:2001 for the detection and isolation of *E. coli* O157 and the VIDAS® *E. coli* O157 system (BioMerieux) according to the manufacturer’s instructions. Strains were further confirmed for the presence of verocytotoxin genes (*vtx1* and *vtx2*), the intimin gene (*eae*) and the enterohemolysin gene (*ehxA)* using the GeneDiscCycler Instruments (Pall Genesystems, France) according to the manufacturer’s instructions.

*E. coli* O157:H7 isolated from stool samples of cases was compared to *E. coli* O157:H7 isolated from food products and a carcass swab using pulsed field gel electrophoresis (PFGE) and insertion sequence 629-printing (IS629-printing). IS629-printing is a rapid genotyping method based on the variable presence of insertion-sequence 629 throughout the *E. coli* O157:H7 genome described by Ooka et al. [15]. Pulsed field gel electrophoresis was conducted as described in the PulseNet protocol for *E. coli* O157.

## Results

### Course of the outbreak

A first case was reported to the department of public health of Limburg on June 4. On June 5 two other cases from a single household were reported. By June 6 seven cases had been reported. Two days later, June 8, the FASFC conducted a first investigation. Samples from meat products were taken in shops frequently mentioned during exploratory interviews with cases. The meat products were traced back to a slaughterhouse and cutting plant, where more samples were taken. The first laboratory results were available on June 14. The case-control study was set-up on June 13 and the invitations to fill in the online survey were distributed on June 14. The results of the case-control study were available on June 18.

### Patient characteristics

**Figure 1:**
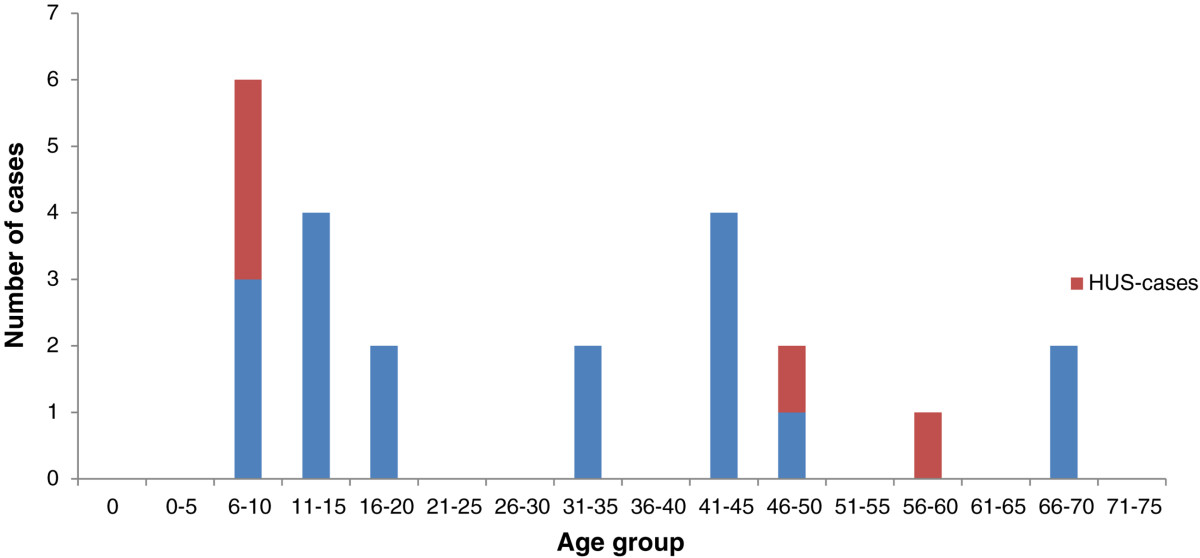
**Age distribution cases.** Age distribution of the cases, Limburg, Belgium 2012.

**Figure 2:**
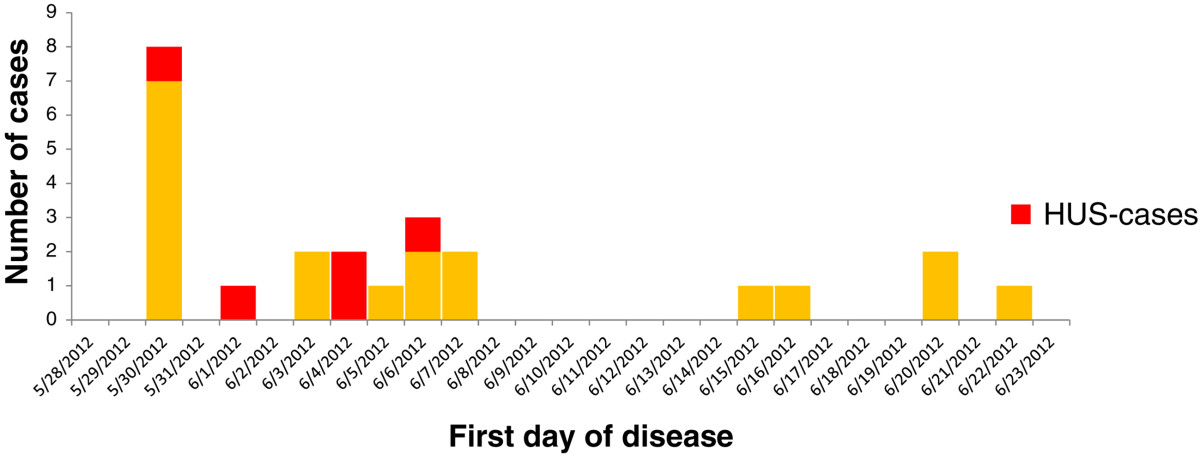
**Epidemic curve.** Epidemic curve, Limburg, Belgium, 2012.

The outbreak involved 24 cases from 17 different households. Seventeen cases were laboratory-confirmed. The seven other cases were family members of laboratory-confirmed cases who reported bloody diarrhea. The outbreak mainly affected children and middle-aged persons. The mean age was 31 years (range: 6-84 years) (Figure 1). Thirteen cases were female, eleven male. Fifteen cases were hospitalized for an average of seven days (range 2-35 days). Five patients developed HUS, three children (two girls, one boy; aged 7, 8 and 10 years) and two adults (female; aged 46 and 58 years). All fully recovered.The first day of disease, as reported by the cases, ranged from May 30 to June 22 (Figure 2). The cases were closely clustered in space. All lived in the North-East region of Limburg, at the most approximately 30 kilometers apart. One case, living in Antwerp, formed an exception but did visit relatives in the affected region. All 17 families were contacted for an exploratory interview. Information on infant cases was provided by a parent. Nine cases named “filet américain” as a possible source. Four cases named “minced beef” as a possible source. The other cases had no specific suspicions (N = 7) or named a different product (N = 4, raw vegetables, sausages, codfish, steak).

### Case-control

Out of the 19 cases known at the start of the case-control study, 13 were laboratory-confirmed and eleven were selected for the case-control study. Out of the 200 persons invited to the survey, 65 did not report any symptoms and completed the survey. They were selected as controls. Out of the 22 food products present in the survey only two were statistically significant in univariate analysis; “ice cream” (OR 8.57, 95% CI 1.03-70.89) and “steak tartare” (OR 48.12, 95% CI 5.62-416.03). Cases were also more likely to shop at, at least one, of the five suspected supermarkets (OR 11.67, 95% CI 1.41, 96.49). The only statistically significant factor in a multivariate regression model was “steak tartare” (OR 41.02, 95% CI 4.51-372.78). A quasi-complete separation of the data did not allow the fitting of a model with an interaction term (“suspected supermarkets”*”steak tartare”) (Table 1).

**Table 1:** **Results from the case-control study, Limburg, Belgium, 2012**

Factor	Odds ratio	Lower 95% CI	Upper 95% CI
**Univariate analysis**			
Suspected supermarkets	11.67	1.41	96.49
Ice Cream	8.57	1.03	70.89
Steak Tartare	48.12	5.62	416.01
**Multivariate analysis**			
Suspected Supermarkets	9.09	0.91	90.65
Steak Tartare	41.02	4.51	372.78

*The following food items were included in the case-control study, but no significant association with cases or controls were found.*

*Beef:* steak, tartare, minced beef, hamburger, tenderloin, roast beef, sausage.

*Pork:* Salami, sausage, minced pork.

*Pork & Beef:* sausage, half beef – half pork minced.

*Vegetables:* sprouts, salad, ready-made salad, crudités, ready-made fruit salad, homegrown vegetables.

*Diary:* raw milk, soft cheese.

### Results of sampling

Food samples were taken at the butcheries of five different supermarkets, one meat establishment, one cutting plant and one slaughterhouse. On June 14, the NRL-FBO confirmed the presence of *vtx1a vtx2a eae ehxA* positive *E. coli* O157 in two samples of raw beef meat and “steak tartare” collected on June 8. Sampling continued until July 4. A total of 73 samples were analyzed by the NRL-FBO. In total, three *vtx1a vtx2a eae ehxA* positive *E. coli* O157 and one *vtx1a vtx2c eae ehxA* positive *E. coli* O157 could be isolated. Ten other samples were suspected for the presence of the epidemic strain at the screening level but the strain could not be isolated due to the presence of a high number of background flora. Five other samples were suspected for the presence of EHEC but the detected virulence genes were different from the ones of the epidemic strain.

**Figure 3:**
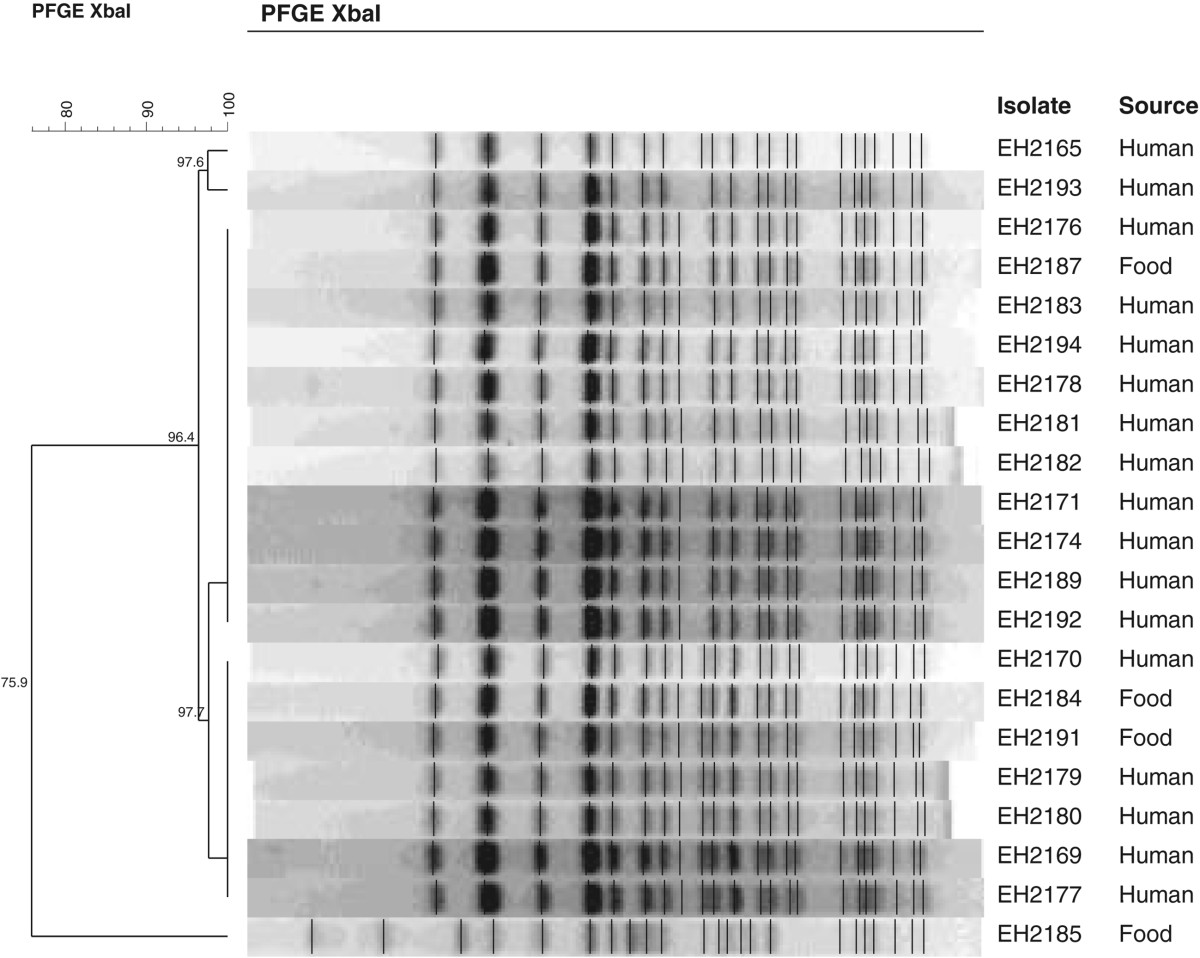
**Molecular analyses.** PFGE of all E.coli O157-strains detected during the outbreak in NE-Limburg. All except EH2185 (vtx1a and vtx2c, carcass swab) are vtx1a vtx2a eae ehxA positive E.coli O157.

All seventeen laboratory-confirmed human cases were positive for *vtx1a vtx2a eae ehxA* positive *E. coli* O157 and had the same IS629-type. Both IS629-printing and PFGE-XbaI demonstrated the clustering of the strains isolated from the human stool samples and three meat products. Except for one isolate from a carcass swab, which was genetically different from the epidemic strain (being a *vtx1a vtx2c eae ehxA* positive *E. coli* strain), no other EHEC-strains were isolated (Figure 3). During the period of the outbreak nine other EHEC-positive stool samples, coming from all over Belgium, were genotyped at the NRC. None were identical to the outbreak type. The 64 human *E. coli* O157-strains isolated by the NRC in 2011 were retrospectively subtyped by IS629-printing. None was of the same type as the outbreak strain. In 2012, prior to the outbreak, this specific strain was isolated twice from human isolates. There was no clear connection between these two isolates and the outbreak. After the outbreak, until 2013, it was not detected again. This strain was not detected in any food products, other than the three meat product involved in the outbreak, from 2011 till 2013.

## Discussion

The management of a foodborne outbreak involves several steps. The outbreak needs to be detected as early as possible, followed by a timely identification of the suspected food vehicle and finally resulting in adequate control measures.

1) Early detection

In Belgium, early identification of an outbreak depends on the alertness and subsequent reporting by clinicians and labs. As also illustrated by this outbreak, a higher alertness is justified during warmer periods. Preoccupied customers, leaving their products in the car, and malfunctioning refrigerators will allow for *E. coli* growth during these periods. *E. coli* grows from 8°C, with an optimal growth at 37°C. Even at 5°C the pathogen remains hazardous [16].

The first cases developed symptoms on May 30. A first case was reported on June 4. On June 8 the first investigation towards the source of the outbreak started. We detected the outbreak relatively fast as compared to other outbreaks, e.g. the *E. coli* O104:H4 outbreak in Germany was only detected two weeks after the outbreak started [17]. Further shortening these intervals will require that clinicians report faster. Public health authorities should focus on an easy and accessible notification system and increase the overall awareness of clinicians about foodborne outbreaks by e.g. frequently reporting about such outbreaks. Outbreak detection by laboratories will be facilitated by the development of new techniques and methods. Molecular information, collected by IS629-printing of every EHEC O157-strain found in clinical samples, is brought into a national database which allows for early detection of outbreaks even in geographically and temporally less obviously associated cases. A database on both national and European level based on PFGE information is in development for EHEC-strains isolated from food.

*E. coli* is a continuously evolving species. New and emerging EHEC-clones will belong to both serogroup O157 and non-O157 serogroups. With the publication of ISO/TS 13136 in November 2012, a standardized method for the detection in foodstuff of other EHEC-serogroups (O26, O103, O111, O145) is available and used at the NRL-FBO. For any *vtx*-positive stool sample multiplex PCR is used to detect EHEC. Peripheral labs may not utilize these more advanced and intensive techniques to detect EHEC. This emphasizes the role of reference labs.

2) Timely identification of the suspected food vehicle

On June 14 the first EHEC-positive meat products were identified. Results from the case-control study were available from June 18. This is 14 and 19 days after the first case experienced symptoms and five and ten days after the first investigation started. Public health authorities communicated for the first time about possible sources of this outbreak 15 days after its onset. The median time to communication by governmental sources for European outbreaks is 31 days [18]. In this outbreak, the main delay was not due to the investigation itself, but due to the time till the detection of the outbreak and the start of the investigation. For the timely identification of the suspected food vehicle, we benefited from the textbook character of this outbreak; contaminated bovine-derived products are the most common source of infection, 75% of EHEC outbreaks are linked to their consumption and a previous Belgian survey showed a prevalence of 0.73% for STEC O157 in beef [19, 20].

A case control study allows for a wide search, it can indicate any item from a survey as possible source, and it can be used to direct microbiological investigations. Studies have been inconclusive about the response representativeness of electronic surveys versus mail surveys, but since an electronic survey is both cheap and fast (in this study there were five days between the set-up of the study and the results) and our population probably had high internet usage, we opted for an electronic survey [21]. After the authorities communicated about the outbreak, the first microbiological results were published in the media [22]. This information was communicated while cases and controls could still fill in the survey, this will likely have interfered with the case-control study. A possible solution to avoid such a possible bias is to use a virtual cohort. This method is based on sales records. It calculates relative risks based on the amount of a certain product sold to cases and the amount sold to persons not identified as cases [23].

A strain identical to the one isolated from human samples was isolated from three meat products. We did not isolate this specific strain from any other sample taken during the trace-back investigation, but we did isolate another EHEC O157 strain from a carcass swab. No environmental sampling was performed in the slaughterhouse, though this could have aided in finding the specific cause (e.g. contaminated during the evisceration process, contaminated equipment and/or uncontrolled environmental conditions) of this outbreak.

3) Control measures and secondary effects

As soon as the presence of *vtx1a vtx2a eae ehxA* positive *E. coli* in meat products was confirmed, the FASFC performed a trace-back investigation using the system of Sanitel numbers. All batches of contaminated beef meat still in retail were put under seizure and destroyed. The butcheries and slaughterhouse were temporarily closed and thoroughly cleaned and disinfected. After the disinfection, butcheries at supermarkets and the slaughterhouse restarted their activities at a slower pace and under close monitoring. The personnel of these establishments was reminded about hand hygiene, cross-contamination and the inspection of animals. They also received a new self-checklist.

Posters and leaflets were distributed at the affected supermarkets. National media coverage of the outbreak aided in publicizing the source of the outbreak and lowering the public anxiety. Still three new cases were reported after national news media reported on the outbreak. These cases kept consuming meat products bought during the outbreak period which were stored in the freezer or refrigerator.

Out of our 24 cases, 5 cases (21%) developed HUS. This amount is relatively high compared to other described EHEC outbreaks, though O157:H7 infections are known to have the strongest association with HUS worldwide [24]. It is known that the use of conventional antibiotics exacerbates Shiga toxin-mediated cytotoxicity [25, 26]. The three children who developed HUS did however not receive antibiotics.

## Conclusion

We described a foodborne outbreak, involving 24 cases of which 5 developed HUS, in which the source could be traced back to a slaughterhouse. PFGE and IS629-printing proved that beef products, sold at different supermarkets, were the source of this EHEC O157:H7 outbreak. Contaminated food products and a contaminated carcass were removed. There was a nine days delay between the start of the outbreak and the start of the investigation. Detecting an outbreak as fast as possible and reporting the results to all concerned are the greatest challenges towards significantly improving outbreak management in Belgium. A lot of different authorities, institutes and laboratories, each with its own mandate, are involved in outbreak investigation and management. An outbreak in which the source is proven and subsequently halted showed the effectiveness of the management of foodborne outbreaks in Belgium.

## Authors’ original submitted files for images

Below are the links to the authors’ original submitted files for images. Authors’ original file for figure 1 Authors’ original file for figure 2 Authors’ original file for figure 3
