# Supplementary figures and images for: Lessons learned from a textbook outbreak: EHEC-O157:H7 infections associated with the consumption of raw meat products, June 2012, Limburg, Belgium
Source: Arch Public Health. 2014 Dec 15;72:44. doi: 10.1186/2049-3258-72-44 (PMC4373035; doi:10.1186/2049-3258-72-44)

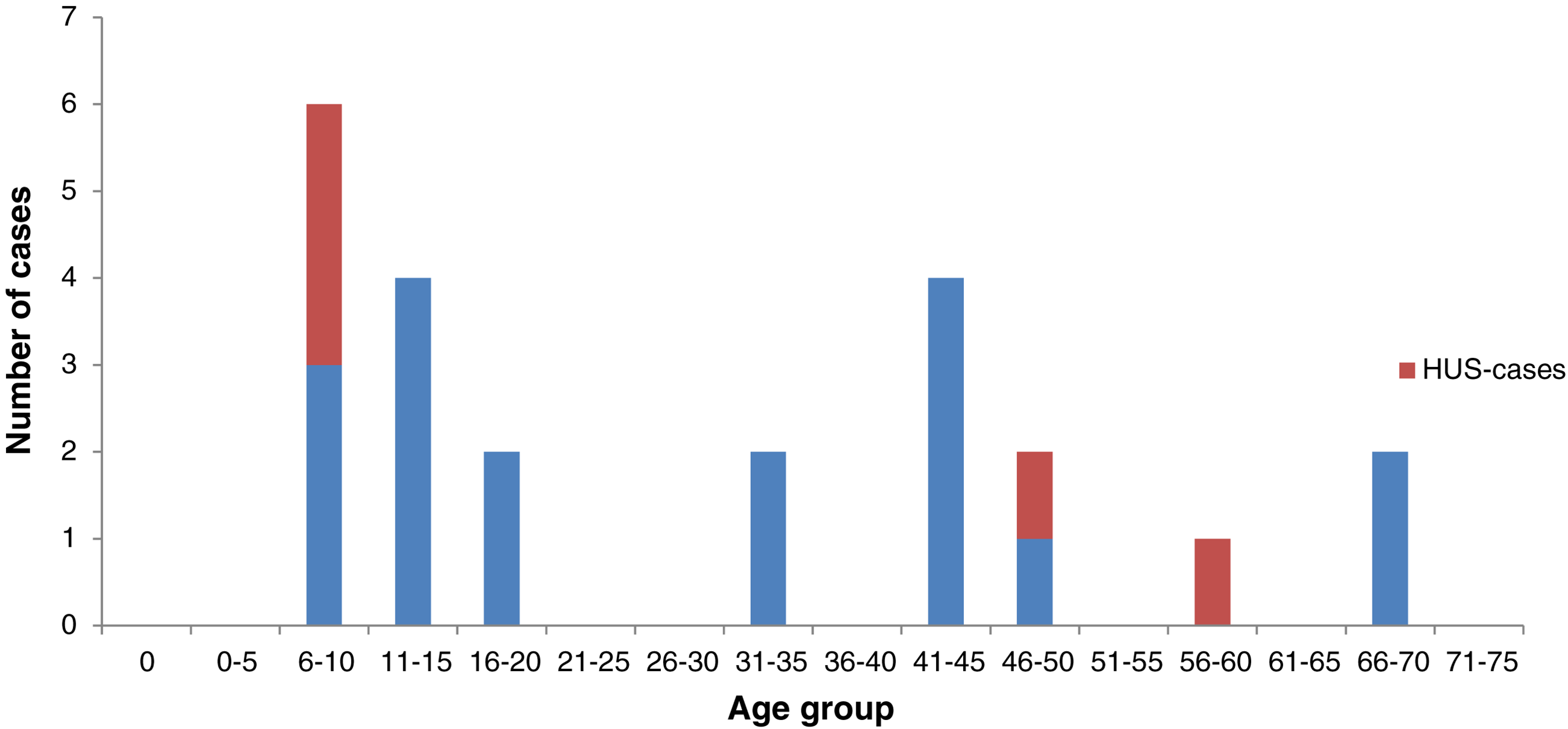

Supplement: Supplementary file 1 — Authors’ original file for figure 1 [file 13690_2014_5063_MOESM1_ESM.tif]

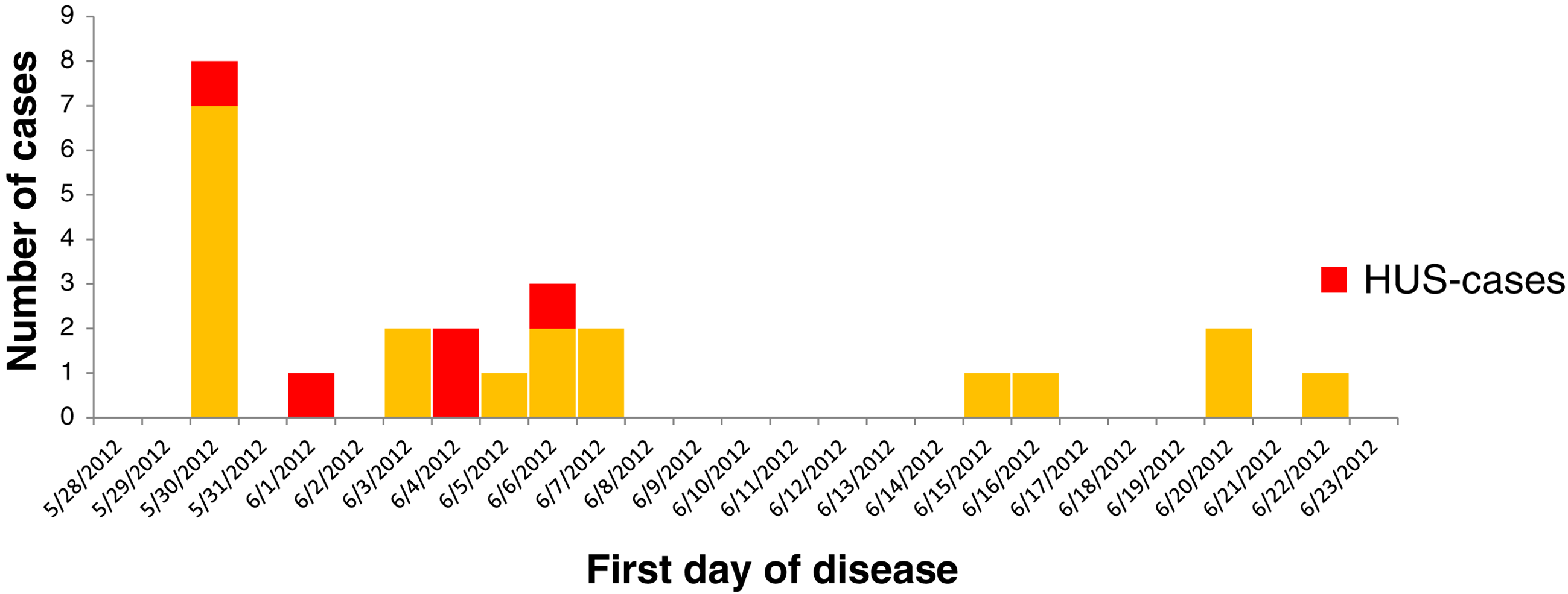

Supplement: Supplementary file 2 — Authors’ original file for figure 2 [file 13690_2014_5063_MOESM2_ESM.tif]

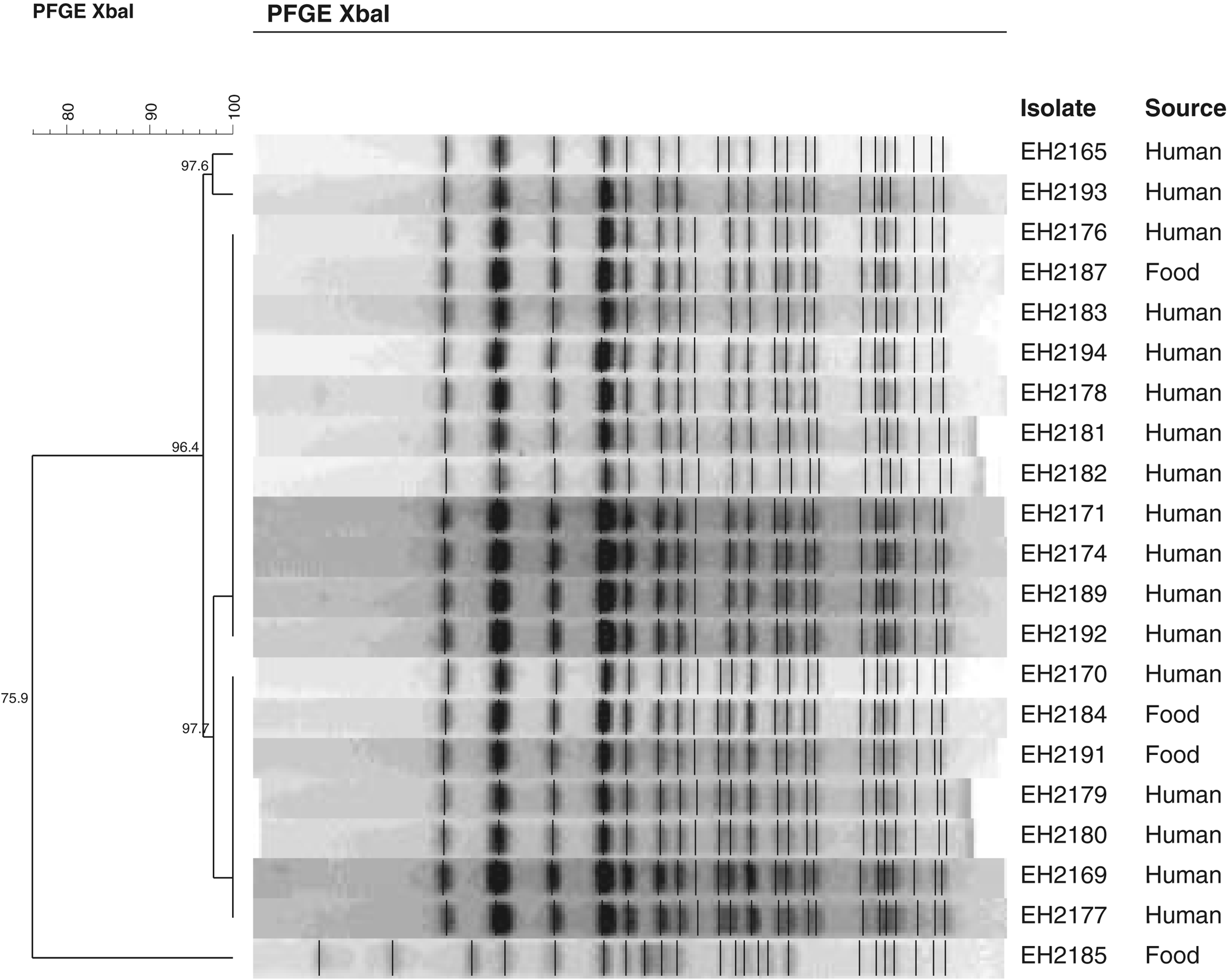

Supplement: Supplementary file 3 — Authors’ original file for figure 3 [file 13690_2014_5063_MOESM3_ESM.tif]
